# Associations Between Platform Leadership and Core Competencies of Novice Nurses in Neurointerventional Departments: The Mediating Role of Achievement Motivation

**DOI:** 10.1155/jonm/9480474

**Published:** 2026-11-15

**Authors:** Guifang Zhang, Zhenya Liu, Shijuan Gao, Xinlan Zhu, Minglu Zuo, Qiuhuan Jiang

**Affiliations:** ^1^ Cerebrovascular Department of Henan Provincial People’s Hospital Henan Provincial Key Medicine Laboratory of Nursing Zhengzhou University People’s Hospital Zhengzhou Henan China; ^2^ Henan Provincial People’s Hospital Henan Provincial Key Medicine Laboratory of Nursing Zhengzhou University People’s Hospital Zhengzhou Henan China hnsrmyy.net

**Keywords:** achievement motivation, core competencies, novice nurses, platform leadership, self-determination theory

## Abstract

**Aim:**

This study seeks to examine the associations between platform leadership and core competencies of novice nurses in neurointerventional departments, utilizing self‐determination theory as a framework, with particular emphasis on the mediating role of achievement motivation.

**Background:**

Core competence is one of the important indicators to guarantee the quality of clinical nursing. Neurology is a complex discipline with diverse lesions, types, symptoms, and treatment methods. This requires neurointerventional nurses to have strong core capabilities. There is a significant relationship between leadership behavior and nurses’ core competencies. Platform leadership, as an emerging leadership style, has garnered attention in recent years. However, research on the relationship between platform leadership and core competencies of novice nurses in neurointerventional departments remains relatively limited.

**Methods:**

This cross‐sectional study recruited 227 novice nurses from neurointervention departments of secondary and tertiary hospitals in Zhengzhou, Henan Province, using convenience sampling. Data were collected via a General Information Questionnaire, Platform Leadership Scale, Achievement Motivation Scale, and Core Competence Questionnaire for neurointervention nurses. All scales demonstrated good reliability and validity. Pearson correlation analysis examined variable correlations, while hierarchical linear regression was adopted to explore factors associated with novice nurses’ core competencies. The SPSS process macro was used to verify the mediating role of achievement motivation between platform leadership and novice nurses’ core competencies.

**Results:**

The score of novice nurses’ core competencies was 169.67 ± 41.61. Core competence was positively correlated with platform leadership (*r* = 0.416, *p* < 0.01) and pursuit of success (*r* = 0.332, *p* < 0.01) and negatively correlated with avoidance of failure (*r* = −0.484, *p* < 0.01). Hierarchical regression analysis showed that hospital grade, monthly income, education level, platform leadership, pursuit of success, and avoidance of failure were significant predictors of novice nurses’ core competence, collectively explaining 49.0% of the variance (adjusted *R* ^2^ = 0.464). Parallel mediation analysis revealed that pursuit of success (indirect effect = 0.2886, 95% CI [0.1345, 0.4672]) and avoidance of failure (indirect effect = 0.3447, 95% CI [0.1515, 0.5678]) significantly mediated the relationship between platform leadership and core competencies. The total indirect effect was 0.6333 (95% CI [0.3739, 0.9264]), accounting for 56.24% of the total effect (*c* = 1.1259, *p* < 0.001).

**Conclusions:**

The core competence score of the novice nurses is not high. Platform leadership, pursuing success, and avoiding failure can independently predict the core competencies of novice nurses; pursuing success and avoiding failure also exert parallel mediating effects between platform leadership and core competencies.

**Implications for Nursing Management:**

Healthcare executives can encourage head nurses to adopt the platform leadership approach to enhance novice nurses’ achievement motivation and take the two dimensions of achievement motivation (pursuing success and avoiding failure) as the intervention targets to enhance the promoting effect of platform leadership on core competence.

## 1. Introduction

Stroke is the second leading cause of death and the third leading cause of both mortality and disability among noncommunicable diseases worldwide, placing a significant burden on society [1]. Data indicate that stroke is the leading cause of disability‐adjusted life years (DALYs) in China, surpassing heart disease, respiratory conditions, and digestive system tumors [2]. Stroke can be classified into ischemic stroke and hemorrhagic stroke. According to the direct reporting data from the China Advanced Stroke Center, in 2020, 635 advanced stroke centers nationwide participated in the data direct reporting system for advanced stroke centers, with a cumulative total of 1,532,109 cases included. Among these, ischemic stroke accounted for 72.72%, intracerebral hemorrhage for 20.46% (including subarachnoid hemorrhage at 5.00%), and other types for 6.82% [3]. In recent years, cerebrovascular interventional surgery has increasingly become a crucial diagnostic and therapeutic approach for cerebrovascular diseases due to its safety, efficacy, and minimal invasiveness. Patients undergoing cerebrovascular interventional procedure therapy need continuous hospitalization and monitoring in the postoperative period to ensure the efficacy of the treatment and to maintain optimal postoperative health status. Nurses within the neurointervention department are required to apply their professional knowledge, advanced technical skills, and clinical judgment comprehensively to manage dynamic postoperative conditions and to prevent life‐threatening complications. The nursing procedures associated with this care demand a high level of specialization, which fully reflects the core competencies essential for delivering high‐quality neurointerventional care [4].

The International Council of Nurses (ICN) first introduced a core competency framework for generalist nurses in 2003 [5], defining these competencies as the ability to integrate and apply knowledge, skills, and clinical judgment in daily practice. Building on this foundation, Liu Ming [4] developed a core competency framework tailored for registered nurses in China based on the ICN’s “Nurse Competency Framework” from 2006. As research has evolved, Chinese scholars have increasingly focused on specialized areas, examining the core competencies of nurses in specific roles, including operating room nurses [6], hemodialysis specialists [7], emergency personnel [8], hospice care providers [9], community health workers [10], and interventional specialists [11]. However, core competencies of novice nurses in neurointerventional departments remain underexplored.

The concept of “from novice to expert” was initially introduced by Benner, who characterized a novice nurse as an individual deficient in authentic clinical practical experience [12]. However, international research has since embraced varying experience thresholds, ranging from 1 to 5 years, to delineate early‐career nurses, thereby reflecting regional differences. In this study, a novice nurse is defined as a registered nurse with less than 2 years of clinical experience, in accordance with the criteria outlined in the Training Outline for Newly Appointed Nurses (Trial) issued by China’s National Health and Family Planning Commission in 2016 [13], and they represent an indispensable new force within the clinical nursing team. As a distinct cohort transitioning into professional practice, novice nurses often find themselves ill‐equipped with the requisite experiential knowledge, technical expertise, and confidence for the clinical environment, a challenge that is compounded by increasing patient demands for specialized care and a growing nursing workload. Research indicates that the core competence of novice nurses is relatively low [14, 15], exhibiting a significant gap when compared to professional standards. The early stages of employment constitute a crucial period during which a nurse’s core competence develops most rapidly [16]. However, few studies have focused on the core competence of novice nurses in the departments of neurointervention in China. Novice nurses in these departments face the dual challenges of transitioning from students to professional practitioners while simultaneously adhering to the highly rigorous demands of the specialty. Enhancing their core competencies therefore requires a more targeted and specialized approach.

In Chinese clinical practice, head nurses serve as both the primary supervisors for novice nurses and frontline leaders who work closely with their nursing teams on a daily basis. The leadership behavior of head nurses can effectively predict nurses’ professional attitudes and behavioral performance [17, 18]. Previous research has indicated that head nurses’ leadership is a significant predictor of nursing staff’s core competencies [19]. However, current research on the factors associated with the core competencies of newly hired nurses in the neurointervention department remains relatively limited. In other specialties, factors associated with the core competence of novice nurses primarily include demographic characteristics (such as age, gender, education, and professional title), participation in relevant training programs (including training duration), involvement in nursing research [15, 20, 21], professional identity [22], job satisfaction, and family support [23]. Research examining these factors remains limited, and deeper‐seated contributing factors require further exploration.

In the context of evolving organizational landscapes and the increasing prevalence of knowledge workers, traditional rigid hierarchical management models within healthcare institutions have progressively weakened, giving way to widespread trends of decentralization and dehierarchization. Within this framework, nurse managers are now required to cultivate equitable, collaborative developmental relationships with frontline nursing staff, leading to the emergence of a novel construct: platform leadership [24]. This concept, characterized as a bottom‐up dynamic management model, seeks to unlock employees’ developmental potential by constructing and continuously refining shared career growth platforms, thereby providing nurses with ample opportunities to demonstrate their professional talents [25]. Unlike traditional unilateral leadership paradigms, platform leadership balances the interests of managers, subordinates, and the entire department, facilitating harmonious team development through mutual tolerance, reciprocal progress, and shared growth. A distinguishing characteristic of this approach is the emphasis placed by leaders on promoting the collective development of subordinates, themselves, and the organization through the creation of a unified platform for collaboration [26]. Its six fundamental dimensions differ significantly from the well‐established paradigms of transformational leadership, empowering leadership, authentic leadership, and inclusive leadership within the realm of nursing management research. Transformational leadership refers to a leader’s ability to significantly influence organizational members through personal charisma, compelling appeal, forward‐looking vision, and motivational strategies, inspiring them to transcend self‐interest and work toward organizational goals [27]. Its core principle is leader‐centricity with a strong focus on organizational objectives, representing a top‐down approach to influence. In contrast, platform leadership places greater emphasis on mutual support and shared growth between leaders and their subordinates; this is the fundamental distinction between platform leadership and transformational leadership [24]. Empowering leadership influences subordinates through power sharing, incentives, and development support, aiming to foster employees’ self‐leadership through one‐way empowerment. The key difference between platform leadership and empowering leadership lies in the fact that platform leadership is based on the establishment of shared platforms that provide subordinates with opportunities to demonstrate their talents [28]. Authentic leadership is a behavioral model that draws upon and fosters positive psychological capabilities and a positive ethical climate to cultivate greater self‐awareness and an internalized moral perspective. Its core lies in the leader’s authenticity, moral character, and transparency, emphasizing personal intrinsic qualities and focusing on ethical and character dimensions, whereas platform leadership emphasizes structural and resource aspects [29]. Inclusive leadership represents a proactive leadership approach that enhances team members’ sense of belonging within the workplace. Inclusive leadership centers on employees, addressing the diverse needs of subordinates, listening to their perspectives, and recognizing their contributions; platform leadership, on the other hand, focuses on a shared mission between leaders and subordinates, guided by the principle of mutual support [28]. Overall, the four leadership styles all employ a unidirectional top‐down logical framework, each focusing on a single dimension: leader charisma, one‐way delegation, personal ethics, and individual employee needs; in contrast, platform leadership stands out by emphasizing shared platforms and a two‐way, mutually beneficial growth mechanism, fundamentally distinguishing it from existing leadership theories in nursing research.

This study utilizes self‐determination theory (SDT) to elucidate the impact of platform leadership on the development of core competencies among novice nurses. Originally proposed by Deci and Ryan, SDT offers a comprehensive framework for understanding human motivation and personality development, highlighting the dynamic interaction between intrinsic growth tendencies and extrinsic environmental factors [30]. SDT posits that an individual’s career motivation development and competence enhancement primarily depend on the continuous fulfillment by the external environment of three fundamental psychological needs: autonomy, competence, and belonging. A supportive external environment can transform external incentives into intrinsic motivation, thereby fostering long‐term positive personal growth [31]. Within the high‐risk, high‐precision working context of neurointerventional care, novice nurses experience prominent role transition challenges and professional adaptation difficulties, which contribute to elevated stress [31]. The fulfillment of their psychological needs directly influences their learning commitment and professional development. This framework comprehensively fosters a departmental environment conducive to newcomer development, addressing fundamental psychological needs such as autonomy, sense of belonging, and competence among novice nurses. According to the motivation internalization mechanism of SDT, the sustained fulfillment of these three psychological needs enables external management resources and training support to transform into stable intrinsic psychological motivation within individuals, ultimately giving rise to structured achievement motivation, precisely the core essence of the achievement motivation concept.

As an internalized and stable psychological tendency, the achievement motive is defined as a relatively stable disposition (or capacity) that manifests as an individual’s pride in accomplishment or hope for success in activities where standards of excellence are applicable [32]. Atkinson [33] proposed the conflict model of “pursue success and avoid failure” of achievement motivation. In an achievement‐oriented context, individuals’ motivational tendencies toward success and fear of failure become activated. The intensity of achievement motivation is determined by the discrepancy between these two tendencies. According to Atkinson [33, 34], the achievement motivation of nurses refers to the intrinsic drive within individuals that compels them to exert their utmost effort toward accomplishing goals during the goal‐completion process. Wang et al. [34] discovered that novice nurses exhibited a low achievement motivation score, characterized by a pronounced tendency to avoid failure in their work. When nurses possess low achievement motivation, their goals tend to be relatively modest, and they are less inclined to exert additional effort to enhance their core competencies while completing tasks. Research has demonstrated that nurses’ achievement motivation is a significant positive predictor of their core competence [35]. Smama’h et al. [36] found that the supportive and achievement‐oriented leadership styles exhibited by head nurses enhance the work motivation of nurses.

Therefore, this study introduces achievement motivation as a mediating variable to construct a theoretical model of “platform leadership‐achievement motivation‐core competencies of novice nurses” and employs SDT to examine the associations between platform leadership and core competencies of novice nurses in neurointerventional departments. This research aims to provide a theoretical basis for developing intervention strategies to enhance novice nurses’ core competence in the department of neurointervention.

## 2. Methods

### 2.1. Study Design and Participants

This was a multicenter, cross‐sectional investigation. A self‐report questionnaire was distributed to nurses (*n* = 227) *via* convenience sampling in secondary and tertiary hospitals in Zhengzhou, Henan Province, from November 2024 to February 2025. The inclusion criteria were as follows: ① registered nurses working in departments of neurointervention; ② working time ≤ 2 years [13]; ③ the work unit is either a secondary or tertiary hospital; and ④ volunteered to participate. The exclusion criteria were as follows: ① nurses who were not on duty, such as on vacation or for further study and ② nurses from other hospitals who were visiting and studying in the surveyed hospitals.

According to Kendall’s rough estimation method for sample size [37], the sample size should be 5 to 10 times the number of variables. This study involves 9 variables related to general information, 6 concerning platform leadership, 2 associated with achievement motivation, and 6 linked to core competence, totaling 23 variables. Thus, the required sample size was estimated to range from 115 to 230. Considering a potential 20% invalid response rate, the final sample size should ideally range between 138 and 276. The initial sample size was set at 276. Of the 276 questionnaires distributed, 227 valid responses were received, resulting in an effective response rate of 82.25%.

### 2.2. Measurements

#### 2.2.1. General Information Questionnaire

The questionnaire was developed by the researchers and included nine items, including gender, age, education, working years, professional title, marital status, children, hospital grade, and monthly income.

#### 2.2.2. Platform Leadership Scale

The platform leadership scale developed by Hao Xuguang [24] was used to measure the level of platform leadership perceived by nurses in this paper. The scale contained six dimensions of “tolerance,” “charisma,” “revolution planning,” “platform building,” “platform optimization,” and “mutual growth,” with a total of 25 items. The setting of items is as follows: “My department leader does not mind that his subordinates are better than himself in some aspects,” “My department leader does not mind and often encourages his subordinates to give him suggestions,” and “My department leader encourages his subordinates to constantly seek new ideas and methods when solving problems,” and so on. Likert’s 5‐point scoring method was used in the scale, from “very inconsistent” to “very consistent.” The higher the score, the higher the level of platform leadership perceived by nurses. The scale has been used in nursing studies [38, 39]. In this study, Cronbach’s *α* coefficient of the scale was 0.964, and Cronbach’s *α* coefficient of six dimensions was 0.785–0.870.

#### 2.2.3. Short Form of Achievement Motive Scale (AMS‐SF)

The AMS‐SF revised by Tang Hui [40] was used, which can measure two different concepts of achievement motivation: the motivation for pursuing success and the motivation for avoiding failure. The scale consisted of 12 items, the first 6 items constituted the pursuing success subscale, and the last 6 items constituted the avoiding failure subscale. Each item is scored on a scale of 1 to 4, and the total score is the difference between the score of the pursuing success subscale and the score of the avoiding failure subscale. Cronbach’s *α* coefficients of the pursuing success and avoiding failure subscales in this study were 0.742 and 0.894, respectively. This scale has been used in nursing studies [41].

#### 2.2.4. The Core Competence Scale for Interventional Nurse

The scale was developed by Guo Wenhui [42] of Hunan Normal University in 2016. The scale included six dimensions: clinical professional practice ability, critical thinking ability, interpersonal communication ability, nursing clinical management ability, scientific research ability and guidance ability, with a total of 50 items. Likert’s 5‐point scoring method was used, and “never, occasionally, sometimes, often, always” were assigned 1–5 points respectively. The higher the score, the higher the level of nurses’ core competence. Scoring rate = (actual score/maximum theoretical score) × 100%, and a higher scoring rate means a higher level of core competence. This scale was previously used by our team in a study and showed excellent reliability and validity among nurses [43]. In this study, Cronbach’s *α* coefficient of the scale was 0.972, and Cronbach’s *α* coefficient of the six dimensions ranged from 0.745 to 0.912.

### 2.3. Data Collection

The study used an online questionnaire survey method. Researchers distributed the electronic questionnaire via WeChat to nursing managers in neurointervention departments, who then forwarded it to novice nurses. Before completing the questionnaire, participants were informed of its purpose, the voluntary and anonymous nature of their participation, and the fact that there were no right or wrong answers. After clicking “agree,” participants completed the questionnaire. Access was restricted to one response per WeChat account, and all items were set as required to ensure data completeness and response validity.

### 2.4. Ethical Considerations

Our study received approval from the Ethics Committee of Henan Provincial People’s Hospital (Ethics Code: 2022‐041‐02). All participants were provided with comprehensive information regarding the study and its objectives prior to their inclusion.

### 2.5. Statistical Analysis

Data were analyzed using IBM SPSS Statistics 25.0. Categorical data are presented as frequencies or percentages, while continuous variables are reported as means and standard deviations. Pearson correlation analysis was employed to assess associations between variables. A hierarchical multiple linear regression analysis was conducted to examine the unique contributions of platform leadership and achievement motivation to novice nurses’ core competencies, after controlling for demographic variables. Mediation analysis was conducted to examine whether the relationship between platform leadership and nurses’ core competencies was mediated by pursuing success and avoiding failure dimensions. We tested a parallel mediation model using the SPSS PROCESS macro (Model 4), with 5000 bootstrap samples. The point estimate of the indirect effect and its 95% bias‐corrected bootstrap confidence interval (CI) were calculated. An indirect effect was considered statistically significant if the corresponding CI did not include zero. A two‐tailed *p* < 0.05 was considered statistically significant.

This cross‐sectional observational study was reported in accordance with the Strengthening the Reporting of Observational Studies in Epidemiology (STROBE) statement for cross‐sectional research [44].

## 3. Results

### 3.1. Demographics

Among the 227 newly employed nurses in the neurointerventional departments surveyed, their ages ranged from 19 to 31, with a mean age of 24.40 ± 2.65 years and all of them were female. Working years ranged from 0.5 to 2 years, with a mean of 1.52 ± 0.57 years. A summary of the sample characteristics is provided in Table 1.

**TABLE 1:** Sample demographics (*N* = 227).

Category		*N* (%)
Gender	Male	0 (0)
	Female	227 (100)

Professional title	Primary title	214 (94.3)
	Intermediate title	13 (5.7)
	Senior title	0 (0)

Education	Junior college	138 (60.8)
	Bachelor’s	75 (33.0)
	Masters and above	14 (6.2)

Working years (years)	0.5	22 (9.7)
	1	77 33.9)
	2	128 (56.4)

Marital status	Married	55 (24.2)
	Single	172 (75.8)

Children	No	188 (82.8)
	One	39 (17.2)

Hospital grade	Secondary hospital	108 (47.6)
	Tertiary hospital	119 (52.4)

Monthly income (¥)	< 3000	84 (37.0)
	3000–5000	97 (42.7)
	5000–7000	46 (20.3)

### 3.2. Common Method Bias Test

Common method bias was examined using Harman’s single‐factor test. Unrotated exploratory factor analysis was conducted for all questionnaire items. The first extracted common factor explained 36.91% of the total variance, which was lower than the critical threshold of 40%. This result indicated that serious common method bias was not present in the current study.

### 3.3. Means, Standard Deviations and Correlations of Study Variables

Table 2 showed that platform leadership had a mean score of 98.39 (SD = 15.39), pursuing success had a mean of 17.71 (SD = 3.50), avoid failure had a mean of 18.47 (SD = 3.86), and core competencies had a mean of 169.67 (SD = 41.61). The scoring rates across the six dimensions of core competence ranged from 66.03% to 69.89%. The correlation between core competencies and platform leadership (*r* = 0.416), pursuing success (*r* = 0.332), avoiding failure (*r* = −0.484) was significant (*p* < 0.01).

**TABLE 2:** Means, standard deviations, and correlations of study variables.

	Score	SD	1	2	3	4
1. Platform leadership	98.39	15.39	1			
2. Pursuit of success	17.71	3.50	0.455^∗∗^	1		
3. Avoidance of failure	18.47	3.86	−0.303^∗∗^	−0.035	1	
4. Core competencies	169.67	41.61	0.416^∗∗^	0.332^∗∗^	−0.484^∗∗^	1

^∗∗^ *p* < 0.01.

### 3.4. Hierarchical Multiple Linear Regression Predicting Core Competencies of Novice Nurses

Hierarchical multiple regression analysis was conducted to examine the unique contribution of platform leadership and achievement motivation to novice nurses’ core competencies, with demographic variables controlled (Table 3). Model 1 included control variables (education level, hospital grade, monthly income, neurointerventional experience, age, professional title, marital status, and having children), Model 2 added platform leadership, and Model 3 added pursuit of success and avoidance of failure. All variance inflation factor values were below 3.1, indicating no multicollinearity concerns.

**TABLE 3:** Hierarchical multiple linear regression predicting core competencies of novice nurses (*N* = 227).

Variables	Model 1	Model 2	Model 3
Control variables			
Education level	0.239^∗∗^	0.188^∗∗^	0.209^∗∗∗^
Hospital grade	0.258^∗∗∗^	0.238^∗∗∗^	0.161^∗∗^
Monthly income	−0.179^∗∗^	−0.150^∗^	−0.170^∗∗^
Neurointerventional experience (years)	−0.116	−0.110	−0.132^∗^
Age	0.049	0.063	0.089
Professional title	0.021	0.028	0.008
Marital status	−0.081	−0.142	−0.160
Having children	0.133	0.178	0.156
Independent variable			
Platform leadership (PTLD)		0.379^∗∗∗^	0.142^∗^
Mediators			
Pursuit of success (ZQCG)			0.269^∗∗∗^
Avoidance of failure (BMSB)			0.383^∗∗∗^
*R* ^2^	0.188	0.327	0.490
Adjusted *R* ^2^	0.158	0.299	0.464
Δ*R* ^2^	—	0.139^∗∗∗^	0.163^∗∗∗^
*F*	6.295^∗∗∗^	11.716^∗∗∗^	18.814^∗∗∗^

*Note:* Values are standardized regression coefficients (beta). PTLD = platform leadership; ZQCG = pursuit of success; BMSB = avoidance of failure. All variance inflation factor values were below 3.1, indicating no multicollinearity concerns. Categorical variables were coded as follows: education level (junior college = 1, bachelor’s = 2, master’s or above = 3); hospital grade (secondary = 0, tertiary = 1); professional title (primary = 1, intermediate = 2); marital status (unmarried = 0, married = 1); having children (no = 0, yes = 1).

^∗^ *p* < 0.05.

^∗∗^ *p* < 0.01.

^∗∗∗^ *p* < 0.001.

In Model 1, demographic variables accounted for 18.8% of the variance in core competencies (*R* ^2^ = 0.188, *F* = 6.295, *p* < 0.001), with education level (*β* = 0.239, *p* < 0.01), hospital grade (*β* = 0.258, *p* < 0.001), and monthly income (*β* = −0.179, *p* < 0.01) emerging as significant correlates.

In Model 2, the addition of platform leadership significantly increased the explained variance to 32.7% (Δ*R* ^2^ = 0.139, *p* < 0.001), with platform leadership demonstrating a strong standardized coefficient (*β* = 0.379, *p* < 0.001).

In Model 3, the inclusion of pursuit of success and avoidance of failure further increased *R* ^2^ to 0.490 (Δ*R* ^2^ = 0.163, *p* < 0.001). The beta coefficient of platform leadership decreased from 0.379 in Model 2 to 0.142 (*p* < 0.05), suggesting partial mediation by achievement motivation. Pursuit of success (*β* = 0.269, *p* < 0.001) was positively associated, while avoidance of failure (*β* = −0.383, *p* < 0.001) was negatively associated with core competencies. Given the cross‐sectional design, these associations should be interpreted as statistical mediation patterns consistent with the hypothesized model, rather than as evidence of causal pathways.

### 3.5. Parallel Mediation Effect of Pursuing Success and Avoiding Failure

The mediation analysis results (Figure 1 and Table 4) demonstrated significant pathways between platform leadership and novice nurses’ core competencies. The total effect was substantial (*B* = 1.1259, 95% CI [0.8029, 1.4489]), with platform leadership exerting a direct effect (*B* = 0.4926, 95% CI [0.1533, 0.8319]). Partial mediation emerged through two distinct mechanisms: (1) the pursuing success pathway accounted for 25.62% of the total effect (*B* = 0.2886, 95% CI [0.1345, 0.4672]) and (2) the avoiding failure pathway contributed 30.62% (*B* = 0.3447, 95% CI [0.1515, 0.5678]). Collectively, these dual mediation pathways explained 56.24% of the total observed effect.

**FIGURE 1:**
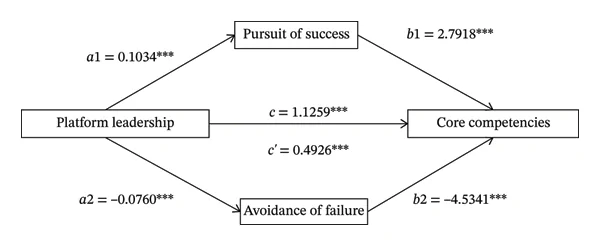
Parallel mediation model presenting path coefficients linking platform leadership to core competencies among novice nurses via two dimensions of achievement motivation (*N* = 227). Coefficient c represents the total effect between platform leadership and core competencies, and c′ indicates the direct effect after controlling for pursuit of success and avoidance of failure. Values shown in the model are unstandardized regression coefficients. ^∗∗∗^ *p* < 0.001.

**TABLE 4:** Mediation analysis of platform leadership and core competencies (*N* = 227).

Model pathways		Estimate	SE	95% CI		Effect proportion (%)
				Lower	Upper	
Direct effect		0.4926	0.1722	0.1533	0.8319	43.75%

Indirect effect	X ⟶ M1 ⟶ Y	0.2886	0.0839	0.1345	0.4672	25.63%
	X ⟶ M2 ⟶ Y	0.3447	0.1084	0.1515	0.5678	30.62%
	Total indirect effect	0.6333	0.1409	0.3739	0.9264	56.25%

Total effect		1.1259	0.1639	0.8029	1.4489	

*Note:* Estimate, unstandardized regression coefficients (B). Platform leadership = X; pursuit of success = M1; avoidance of failure = M2; core competencies = Y.

Abbreviations: CI, confdence interval; SE, standard error.

## 4. Discussion

Given that patients require continued hospitalization following cerebrovascular interventional procedures, nurses specializing in interventional neuroradiology must demonstrate clinical competencies aligned with postprocedural care demands. This study underscores the critical importance of novice nurses’ core competencies in this specialty. Our findings reveal that novice nurses in neurointerventional departments exhibited suboptimal core competencies. Platform leadership was significantly and positively correlated with core competencies, and it was statistically associated with core competencies through two parallel indirect pathways via pursuit of success and avoidance of failure. These findings offer a novel theoretical perspective for understanding the mechanisms underlying core competency development among novice nurses in this specialized clinical context, and provide empirical evidence to inform the design of targeted interventions by nursing managers aimed at enhancing workforce competence in high‐risk neurointerventional settings.

Our study revealed suboptimal core competency levels among novice nurses in interventional neuroradiology, with a core competency score rate of 67.87% (169.67/250). This indicates that, based on the scale standard employed in this study, approximately one‐third of the key competency requirements were not met. What is more concerning is that this performance was lower than that reported for generalist novice nurses [45] and for interventional cardiac nurses [46], highlighting a significant gap between the current skill levels of neurointerventional novice nurses and the demands of this highly specialized, high‐risk clinical domain. Such deficits have direct implications for nursing quality, patient safety, and postoperative health outcomes. The core competency assessment tool employed in this study is a specialized scale specifically designed for interventional department nurses [42], providing a reasonably accurate reflection of competency levels in this population. The score rates across all six competency dimensions ranged from 66.03% to 69.89%, indicating relatively suboptimal performance across all areas and necessitating targeted improvement efforts. The reasons for this gap can be summarized as follows: ① A fundamental cause lies in the theory–practice gap. Novice nurses receive undergraduate education emphasizing foundational competencies but lack sufficient dedicated training for the highly specialized, technology‐intensive demands of neurointerventional nursing. ② The specialized standardized training for novice nurses may be insufficient in depth, breadth, practical operation and duration, which fails to effectively bridge the gap between theory and practice. ③ Novice nurses transition directly into clinical practice following a brief period of training or instruction, yet they often lack access to continuous, stable, and high‐quality on‐the‐job guidance, feedback, and mentorship. ④ Transition shock are common in novice nurses [47], the high‐pressure, technologically demanding environment impedes the pace and efficacy of core competency development. These findings highlight the urgent need to enhance the core competencies of novice nurses in neurointerventional departments. Nurse managers in this specialty can provide crucial support for novice nurses′ core competency development by implementing systemic specialty training programs [43], establishing effective mentorship mechanisms [48] and so on.

Our correlational analyses revealed that platform leadership was positively associated with pursuit of success (*r* = 0.455, *p* < 0.01) and core competencies (*r* = 0.416, *p* < 0.01), and negatively associated with avoidance of failure (*r* = −0.303, *p* < 0.01). These findings suggest that platform leadership, through its six core dimensions (inclusivity, appeal, platform development, platform optimization, transformative planning, and shared growth) systematically addresses novice nurses’ basic psychological needs for autonomy, competence, and relatedness, as conceptualized by SDT [30]. Specifically, inclusivity and appeal support the need for autonomy by respecting individual differences and granting appropriate learning discretion; platform development, optimization, and transformative planning foster competence through structured training, tiered guidance, and continuous feedback; and shared growth cultivates relatedness by promoting reciprocal, supportive relationships between managers and novices. According to SDT, when these three psychological needs are consistently satisfied, externally driven learning behaviors gradually internalize as personally endorsed intrinsic motivation‐a stable psychological tendency that forms the foundation of achievement motivation. This internalized drive, in turn, enables novice nurses to proactively engage in professional development, ultimately enhancing their core competencies [49].

Hierarchical regression analysis revealed that even after controlling for demographic variables (such as educational background, hospital tier, monthly income, etc.), platform leadership remained significantly predictive of novice nurses ′core competencies (*β* = 0.379, *p* < 0.001, Δ*R* ^2^ = 0.139), indicating that the contribution of platform leadership is independent of individual background characteristics. According to SDT, this unique contribution of platform leadership stems from its ability to simultaneously satisfy the three fundamental psychological needs of newly hired nurses—autonomy, competence, and belonging; this mechanism cannot be explained solely by demographic variables. The results of this study show that the *β* coefficient for platform leadership decreased from Model 2 to Model 3, representing a reduction of 62.5%. These findings support the mediating role of achievement motivation, demonstrating that the association between platform leadership and core competencies is primarily mediated by achievement motivation. Furthermore, the relationship between platform leadership and core competencies is largely transmitted through a dual‐channel mechanism involving achievement motivation: the pursuit of success (*β* = 0.269, *p* < 0.001) positively drives proactive learning, while avoiding failure (*β* = 0.383, *p* < 0.001) indirectly promotes competency development by alleviating fear of failure. This pattern indicates that when platform leadership satisfies the basic psychological needs of novice nurses, externally driven learning behaviors gradually internalize into intrinsic achievement motivation, thereby driving the enhancement of core competencies. Therefore, nursing managers can enhance the core competencies of novice nurses by cultivating a platform leadership style among head nurses. For novice nurses, an assessment of their achievement motivation should be conducted. For those with a high motivation to pursue success, core competency improvement projects can be developed based on their training needs [50], or effective core competency training models can be directly selected for their training [51]. For those with a high motivation to avoid failure, nonpunitive safety error analysis training should be provided, and error‐tolerant simulation scenarios should be established [52].

The core finding of this study is that the association between platform leadership and the core competencies of novice nurses in neurointervention is not only characterized by a direct effect but also operates indirectly through two parallel and independent mediating pathways, accounting for 56.25% of the total effect. Specifically, the pathways of “striving for success” (accounting for 25.63% of the total effect) and “avoiding failure” (accounting for 30.62% of the total effect) function independently yet coexist synergistically, collectively constituting a comprehensive motivational framework through which platform leadership influences core competencies (the pathway diagram is shown in Figure 1). Notably, the indirect effect of the “avoiding failure” pathway (30.62%) is slightly higher than that of the “striving for success” pathway (25.63%), suggesting that in the high‐risk, low‐tolerance clinical setting of neurointervention, alleviating novice nurses’ fear of failure may exert a stronger promoting effect compared to stimulating their desire for success. This finding does not indicate any inhibitory effect of one pathway on the other; rather, it reveals a synergistic interaction between the two pathways. This result aligns well with the “zero‐tolerance” clinical characteristics of the neurointervention specialty and provides empirical support for nursing administrators to prioritize the cultivation of psychological safety among novice nurses during training programs. This discovery enriches the theoretical model regarding the relationship between platform leadership and core competencies, particularly for the complex and high‐risk training environment faced by novice nurses in neurointervention departments. This finding accurately reflects the dual psychological state that novice nurses may experience in clinical settings: simultaneously harboring both a “desire to perform well” and a “fear of procedural errors.” At the clinical practice level, platform leadership exerts its influence precisely through these mechanisms. The head nurse, guided by a shared mission, provides resource and platform support [53] to address errors made by new nurses in a constructive manner—by offering timely feedback after they have learned new techniques or managed complex cases, acknowledging their efforts, and reinforcing their incremental progress and successful experiences during skill acquisition; this approach effectively alleviates new nurses′ fear of failure, while simultaneously enhancing their self‐efficacy and intrinsic motivation, thereby motivating them to actively participate in various competency enhancement activities [54], thus transforming the structural advantages of platform leadership into a sustained driving force for professional growth.

Based on these findings, this study proposes the following practical recommendations. Regarding educational initiatives, the platform leadership principles should be integrated into standardized training programs for newly hired nurses through the adoption of modular and tiered training content, the customization of individualized learning paths based on personal differences, and the granting of appropriate autonomy—thereby simultaneously addressing both the self‐directed needs and competency development requirements of new nurses. Concerning the mentorship model, the mentorship approach should transition from the traditional “one‐way knowledge transfer” to a “two‐way collaborative partnership,” encouraging new nurses to actively pose questions and participate in decision‐making, while mentors focus on providing constructive feedback rather than judgmental evaluations; this reciprocal relationship embodies the core essence of “shared growth” inherent in platform leadership. In terms of leadership development interventions, nurse manager training programs should systematically incorporate the six dimensions of platform leadership into their curriculum, with a particular emphasis on cultivating specific managerial behaviors such as “inclusive diversity,” “platform facilitation,” and “promoting collective team development.” Regarding organizational policies, performance evaluation and promotion criteria for head nurses at the hospital level should include platform leadership assessment indicators, elevating this leadership concept from “individual traits” to “organizational expectations.” Furthermore, for new nurses with high avoidance tendencies toward failure, it is recommended to implement nonpunitive safety incident analysis training and fault‐tolerant simulation scenario exercises, which can help them transform their avoidance tendencies into motivation for prudent practice within a safe environment.

From a theoretical perspective, this finding extends the view within Atkinson’s Achievement Motivation Theory—which posits that “striving for success” and “avoiding failure” constitute two independent psychological dimensions—from the individual differences level to the leadership mechanism level, revealing how external leadership behaviors influence behavioral outcomes by simultaneously activating both the “approach” and “avoidance” motivational systems, thereby enriching the application model of platform‐based leadership theory in complex, high‐risk nursing training contexts. We explicitly propose that head nurse training programs should incorporate all six dimensions of platform‐based leadership into their curriculum system, with particular emphasis on specific managerial behaviors such as “inclusive diversity,” “platform facilitation,” and “promoting collective team development.”

## 5. Conclusions

In this study, novice nurses’ core competency scores fell below the desired level. This study found that platform leadership exhibits a significant correlation with novice nurses’ core competency scores, and also functions as direct and indirect statistical associations through two parallel achievement motivation dimensions: pursuit of success and avoidance of failure. These correlational patterns provide a novel analytical perspective for exploring ways to elevate novice nurses’ core competencies and strengthen the theoretical value of this research. Nursing managers should cultivate and improve head nurses’ platform leadership level and attribute significance to the important role of novice nurses’ achievement motivation.

## 6. Limitations

Due to time and resource constraints, this study employed convenience sampling and used self‐assessment tools to survey only 227 novice nurses in neurointerventional departments. This method has several significant limitations in terms of result interpretation: (1) Convenience sampling may introduce significant selection bias; although all hospitals included in this study had implemented the “Training Outline for Newly Appointed Nurses (Trial)” issued by the National Health and Family Planning Commission, all participating medical institutions were secondary and tertiary hospitals in Henan Province; however, the disparities in medical resources, nursing management models, and clinical workload across other provinces mean that this sample did not fully represent all newly appointed nurses working in the field of neurointerventional medicine nationwide, thereby limiting the external generalizability of the findings from this study. (2) The study collected data using self‐administered questionnaires, and the results exhibited subjective reporting bias. (3) This study was a cross‐sectional investigation, which could only verify the correlations among variables and could not establish causal temporal relationships between platform leadership, achievement motivation, and core competencies of novice nurses. (4) The study sample was confined to a single province within China. Regional culture and departmental management environments may influence nurses’ psychological perceptions and behavioral outcomes, suggesting that the findings may exhibit cultural specificity. Longitudinal studies or randomized controlled trials are needed to further validate our findings. As our study relied primarily on quantitative methods, future research could employ qualitative or mixed methods to examine the complex interrelations, and future studies should adopt more objective assessment methods to enhance the reliability of the outcomes.

## Author Contributions

Guifang Zhang: conceptualization, methodology, formal analysis, investigation, data analysis and management, writing–original draft, visualization, and validation; Zhenya Liu: formal analysis, writing–review and editing, validation, and visualization; Shijuan Gao: investigation, resources, and data curation; Xinlan Zhu: investigation, data collection, resources, and validation; Minglu Zuo: conceptualization, methodology, data collection, and writing–review and editing; Qiuhuan Jiang: supervision, project administration, and review.

## Funding

This work was supported by the Henan Province Medical Education Research Project Fund (WJLX2024025).

## Disclosure

All the authors have read and agreed to the final submitted version of this manuscript.

## Conflicts of Interest

The authors declare no conflicts of interest.

## Supporting Information

Additional supporting information can be found online in the Supporting Information section.

## Supporting information


**Supporting Information** STROBE Checklist.

## Data Availability

The raw questionnaire datasets generated and analyzed in this study are not publicly available due to hospital privacy regulations and protection of participants’ personal information, but deidentified datasets are available from the corresponding author upon reasonable written request.

## References

[1] Feigin V. L. , Brainin M. , Norrving B. et al., World Stroke Organization: Global Stroke Fact Sheet 2025, International Journal of Stroke. (2025) 20, no. 2, 132–144, 10.1177/17474930241308142. PMC11786524 39635884

[2] Gbd 2019 Stroke Collaborators , Stark B. A. , Johnson C. O. et al., Global, Regional, and National Burden of Stroke and Its Risk Factors, 1990–2019: A Systematic Analysis for the Global Burden of Disease Study 2019, The Lancet Neurology. (2021) 20, no. 10, 795–820, 10.1016/S1474-4422(21)00252-0. PMC8443449 34487721

[3] Report on Stroke Prevention and Treatment in China Writing Group and Wang L. D. , Brief Report on Stroke Prevention and Treatment in China, Journal of Cerebrovascular Diseases. (2023) 20, no. 11, 783–792, 10.3969/j.issn.1672-5921.2023.11.009.

[4] Liu M. , Kunaiktikul W. , Senaratana W. et al., Framework of the Chinese Registered Nurse’s Competency: a Qualitative Research[In Chinese], Chinese Journal of Nursing. (2006) 41, no. 8, 691–694.

[5] International Council of Nurses , ICN Framework of Competencies for the Generalist Nurse: Report of the Development Process and Consultation, 2003, International Council of Nurses, Geneva, Switzerland.

[6] Liu B. L. , Wei W. X. , Wu B. et al., Research Status of Core Competencies of Operating Room Specialist Nurse[In Chinese], Chinese General Practice Nursing. (2024) 22, no. 14, 2617–2619, 10.12104/j.issn.1674-4748.2024.14.010.

[7] Yu H. Y. , Xiao J. R. , Yang S. R. et al., Research Progress on Evaluation and Training of Core Competence of Blood Purification Specialist Nurses, Chinese General Practice Nursing. (2021) 19, no. 14, 1890–1893, 10.12104/j.issn.1674-4748.2021.14.005.

[8] Chen X. J. and Zhao J. H. , Research Progress on the Core Competence of Nurses in Emergency Department, International Journal of Nursing. (2018) 37, no. 4, 444–446, 10.3760/cma.j.issn.1673-4351.2018.04.003.

[9] Zhao J. , Zhang Y. , Lin X. P. et al., Research Progress on Assessment Tools for Core Competence of Hospice Care Nurses, Chinese Nursing Research. (2020) 34, no. 16, 2896–2900, 10.12102/j.issn.1009-6493.2020.16.017.

[10] Shi Y. L. , Zhang Y. , Liu M. et al., Research Progress on Core Competence of Geriatric Nursing in Community Nurses[In Chinese], Chinese General Practice Nursing. (2022) 20, no. 31, 4380–4385, 10.12104/j.issn.1674-4748.2022.31.014.

[11] Cai Y. M. , Li Y. L. , Tan X. Y. , Guo W. H. , and Ding J. H. , Construction of the Care Competency Evaluation Index for Intervention Specialized Nurses, Journal of Nursing Science. (2016) 31, no. 10, 79–81, 10.3870/j.issn.1001-4152.2016.10.079.

[12] Munjas B. A. , From Novice to Expert: Excellence and Power in Clinical Nursing Practice, Journal of Psychosocial Nursing and Mental Health Services. (1985) 23, no. 5, 10.3928/0279-3695-19850501-10.

[13] National Health and Family Planning Commission of the People’s Republic of China , Training Program for Novice Nurses (Trial), 2016, https://www.2lwecan.com/wsrew/c100280e/202210/1000640.shtml.

[14] Qi H. M. and Zhang G. Q. , The New Nurses’ Self-Esteem Status and Impact on Professional Core Competence, Health Vocational Education. (2025) 43, no. 10, 113–116, 10.20037/j.issn.1671-1246.2025.10.30.

[15] Wang X. L. , Liu L. Y. , Zhang C. H. et al., Investigation on Status Quo of the Core Competency of Newly Recruited Nurses and Analysis of Influencing Factors, Chinese Nursing Research. (2021) 35, no. 8, 1480–1483, 10.12102/j.issn.1009-6493.2021.08.031.

[16] Ou J. N. , Review of Core Competencies of Nurses, Journal of Nursing Science. (2016) 31, no. 3, 98–101, 10.3870/j.issn.1001-4152.2016.03.098.

[17] Lee S. E. and Seo J. K. , Effects of Nurse Managers′ Inclusive Leadership on Nurses′ Psychological Safety and Innovative Work Behavior: the Moderating Role of Collectivism, Journal of Nursing Scholarship. (2024) 56, no. 4, 554–562, 10.1111/jnu.12965. 38467584

[18] Suliman M. , Aljezawi M. , Almansi S. et al., Effect of Nurse Managers′ Leadership Styles on Predicted Nurse Turnover, Nursing Management. (2020) 27, no. 5, 20–25, 10.7748/nm.2020.e1956. 32662259

[19] Gao A. N. , He J. H. , Li S. et al., The Effects of Manager Care and Head Nurse Leadership on the Core Competence of Obstetric Nurses, Anhui Medical Journal. (2024) 45, no. 5, 640–643, 10.3969/j.issn.1000-0399.2024.05.022.

[20] Lai J. , Wen G. , and Gu C. , The Core Competencies in Disaster Nursing of New Graduate Nurses in Guangdong, China: A Cross-Sectional Study, Nurse Education in Practice. (2024) 77, 10.1016/j.nepr.2024.103987. 38678869

[21] Yang F. , Li M. , and Duan H. L. , Study on Present Situation and Influencing Factors of Nurses′ Core Competence in the Operation Room, Chinese Journal of Nursing Education. (2018) 15, no. 7, 526–529, 10.3761/j.issn.1672-9234.2018.07.011.

[22] Wei L. , Cai S. , Mo M. M. et al., Research on Core Competency and Influencing Factors of Standardized Training Period for Newly Recruited Nurses in Traditional Chinese Medicine Hospital, Chinese Journal of Modern Nursing. (2020) 26, no. 20, 2809–2813, 10.3760/cma.j.cn115682-20190612-02096.

[23] Zhang J. W. , Hu X. W. , and Zhang W. , Meta Analysis of Core Competence of Junior Nurses and Its Influencing Factors, Chinese Journal of Modern Nursing. (2022) 28, no. 33, 4632–4638, 10.3760/cma.j.cn115682-20220810-03891.

[24] Hao X. G. and Zhang J. Q. , Platform Leadership: A New Type of Leadership, Beijing Daily. (2021) https://news.uibe.edu.cn/info/1371/45054.htm.

[25] Hao X. G. , Zhang J. Q. , Lei Z. Q. et al., Platform-Based Leadership: Multi-Dimensional Structure, Measurement, and Validation of the Impact on Innovative Behavior, Management World. (2021) 37, no. 1, 186–199, http://dx.chinadoi.cn/10.3969/j.issn.1002-5502.2021.01.012.

[26] Yang X. , Jin R. , and Zhao C. , Platform Leadership and Sustainable Competitive Advantage: the Mediating Role of Ambidextrous Learning, Frontiers in Psychology. (2022) 13, 10.3389/fpsyg.2022.836241. PMC8907919 35282205

[27] Fischer S. A. , Transformational Leadership in Nursing: a Concept Analysis, Journal of Advanced Nursing. (2016) 72, no. 11, 2644–2653, 10.1111/jan.13049. 27322510

[28] Zhang S. , Liu Y. , Li G. , Zhang Z. , and Fa T. , Chinese Nurses′ Innovation Capacity: the Influence of Inclusive Leadership, Empowering Leadership and Psychological Empowerment, Journal of Nursing Management. (2022) 30, no. 6, 1990–1999, 10.1111/jonm.13654. 35476276

[29] Walumbwa F. O. , Avolio B. J. , Gardner W. L. et al., Authentic Leadership Development: Getting to the Roots of Positive Forms of Leadership, The Leadership Quarterly. (2008) 19, no. 1, 89–104, 10.1016/j.leaqua.2007.11.005.

[30] Ryan R. and Deci E. , Self-Determination Theory and the Facilitation of Intrinsic Motivation, Social Development, and well-being, American Psychologist. (2000) 55, no. 1, 68–78, 10.1037/0003-066x.55.1.68. 11392867

[31] Wang S. C. , Shih S. M. , and Kuo L. C. , Exploring Mindfulness Interventions for Stress Resilience in Newly Graduated Nurses: a Qualitative Study, Tzu Chi Medical Journal. (2024) 36, no. 4, 452–459, 10.4103/tcmj.tcmj_250_23. PMC11483087 39421497

[32] Atkinson J. W. , Bastian J. R. , Earl R. W. , and Litwin G. H. , The Achievement Motive, Goal Setting, and Probability Preferences, Journal of Abnormal and Social Psychology. (1960) 60, no. 1, 27–36, 10.1037/h0047990. 13794970

[33] Atkinson J. W. , Motivational Determinants of risk-taking Behavior, Psychological Review. (1957) 64, no. Part 1, 359–372, 10.1037/h0043445. 13505972

[34] Wang Y. , Yang Y. , Gao X. L. et al., Analysis of the Correlation Between Career Anchor and Achievement Motivation of Newly Recruited Operating Room Nurses, Hospital Administration Journal of Chinese People’s Liberation Army. (2020) 27, no. 10, 984–988, 10.16770/J.cnki.1008-9985.2020.10.026.

[35] Feng C. Y. , Relationship Between Achievement Motivation Andcore Competency Among Nurses in Three Tertiarybospitals of Zhuzhou City, China National Knowledge Infrastructure (CNKI). (2015) https://kns.cnki.net/kcms2/article/abstract?v=mV2q5OJ_OLyRNZQrd5knDzZ6CR0btK8ixOt6pEJU8jNbbAJAEpxqknR0nymAXVcHJzGl8z-B-i69fw0uLxgK6aOAWWtQ_1wo-yfgkgFaoIaaw1SKap9VXA==%26uniplatform=CHKD.

[36] Smama′h Y. , Eshah N. F. , Al-Oweidat I. A. , Rayan A. , and Nashwan A. J. , The Impact of Leadership Styles of Nurse Managers on Nurses′ Motivation and Turnover Intention Among Jordanian Nurses, Journal of Healthcare Leadership. (2023) 15, 19–29, 10.2147/JHL.S394601. PMC9884098 36718174

[37] Kendall M. G. and Stuart A. , Kendall′s Advanced Theory of Statistics, 1994, 6th edition, Edward Arnold.

[38] Jiang H. , Chen H. J. , Yang Y. , and Yu L. M. , The Chain Mediating Effect of Perceived Overqualification and Job Crafting on the Relationship Between Platform Leadership and Career Calling of Clinical Nurses, Chinese Journal of Practical Nursing. (2024) 40, no. 5, 350–356, 10.3760/cma.j.cn211501-20230623-01513.

[39] Liu Y. , Zhou H. , and Cao Q. , The Impact of Platform Leadership on Innovative Behavior of Nurses Under the Background of high-quality Development of Public Hospitals, Chinese Journal of Health Policy. (2024) 17, no. 3, 72–78, 10.3969/j.issn.1674-2982.2024.03.010.

[40] Tang H. and Lu N. , Measure Invariance Research of the Short Form of Achievement Motive Scale in Boy-Girl of Middle School Students in Shenzhen, Advances in Psychology. (2013) 03, no. 06, 321–326, 10.12677/AP.2013.36048.

[41] Wang X. Y. , Li C. , Han N. , Wang R. , Liu N. , and Zhang X. L. , Mediating Effect of Achievement Motivation Between Perfectionism and Procrastination Behavior of Hematology Nurses, Chinese Journal of Modern Nursing. (2024) 30, no. 8, 1073–1077, 10.3760/cma.j.cn115682-20230827-00715.C.

[42] Guo W. H. , Development of a Core Competency Assessment Scale for Interventional Specialist Nurses, China National Knowledge Infrastructure (CNKI). (2017) https://kns.cnki.net/kcms2/article/abstract?v=mV2q5OJ_OLzww3XBcKGke01IlwPgx12PdqMdNxLWffbsMDgHr0uHplOkJOmlOIkcBTdBG0DnuNx6Mj3RQZV4qEy684x61EmSssFqVoQ9NEkK_8cQTpRk2w==%26uniplatform=CHKD.

[43] Zhang G. F. , Liu Z. Y. , Gao S. J. , Guo Y. F. , Feng Y. P. , and Zhang H. M. , Design and Application of a Micro-video Training on Core Competence for Nurses in the Interventional Neuroradiology Department, Chinese Journal of Nursing Education. (2022) 19, no. 8, 723–726, 10.3761/j.issn.1672-9234.2022.08.011.

[44] von Elm E. , Altman D. G. , Egger M. , Pocock S. J. , Gøtzsche P. C. , and Vandenbroucke J. P. , The Strengthening the Reporting of Observational Studies in Epidemiology (STROBE) Statement: Guidelines for Reporting Observational Studies, Plos Medinine. (2007) 4, no. 10, 10.1371/journal.pmed.0040296. PMC2020495 17941714

[45] Wang J. , Chen J. , Zheng L. et al., Influence of Psychological Capital on Core Competency for New Nurses, Public Library of Science one. (2023) 18, no. 8, 10.1371/journal.pone.0289105. PMC10414633 37561799

[46] Du M. L. , Zhu L. , Chen S. W. , and Fang L. , Core Competence of Interventional Cardiac Nurses and the Influencing Factors, Journal of Nursing Science. (2023) 38, no. 23, 55–58, 10.3870/j.issn.1001-4152.2023.23.055.

[47] Wang P. , Zhou J. , Shen X. et al., Health Characteristics and Factors Associated with Transition Shock in Newly Graduated Registered Nurses: a Latent Class Analysis, BMC Nursing. (2024) 23, no. 1, 10.1186/s12912-024-01862-8. PMC10976675 38539192

[48] Guo N. , Li Z. , Wang W. et al., Application of dual-tutors Combined with Balint Group in Improving Training Experience and Core Competency of New Nurses, Chinese Nursing Management. (2021) 21, no. 6, 808–812, 10.3969/j.issn.1672-1756.2021.06.003.

[49] Wen S. J. , Hou L. L. , Wu W. H. et al., Mediating Effect of Intrinsic Motivation of Nurses Between Empowering Leadership and Job Performance, Chinese Journal of Practical Nursing. (2025) 41, no. 15, 1168–1175, 10.3760/cma.j.cn211501-20240716-01879.

[50] Zhang J. W. and Zhang W. , Investigation and Analysis of the Current Status of Core Competence and Standardized Training Needs of Newly Recruited Nurses in Cancer Specialized Hospitals, Clinical Research and Practice. (2024) 9, no. 36, 25–28, 10.19347/j.cnki.2096-1413.202436007.

[51] Wu J. R. , Feng J. P. , Zhang L. , and He X. M. , Application of APIE Training Mode in Cultivating Core Competence of Sonior Novice Nurses, Chinese Evidence-Based Nursing. (2024) 10, no. 15, 2792–2795, 10.12102/j.issn.2095-8668.2024.15.025.

[52] Huang H. Y. , Wang Y. F. , Zhou S. , Wang D. , and Yu L. N. , Application of Simulation-based Experiential Learning in Adverse Event Prevention Training for Newly Graduated Nurses, Journal of Nursing Science. (2018) 33, no. 1, 64–65, 10.3870/j.issn.1001-4152.2018.01.064.

[53] Xiong L. , Liu B. , Zhan X. J. , and Zhou Q. , How Does Platform Leadership Enable Knowledge Employees to be Adaptive? A Serial Mediation Model Based on Flow Theory, Journal of Management World. (2023) 39, no. 2, 124–140, 10.19744/j.cnki.11-1235/f.2023.0030.

[54] Hu D. M. , Qin J. Y. , Li C. , and Deng Y. , Platform Leadership and Employee Proactivity: on the Mediating Role of Workplace Thriving and the Moderating Role of Role Breadth Self-Efficacy, West Forum on Economy and Management. (2023) 34, no. 6, 68–78, 10.12181/jjgl.2023.06.07.

